# To the Editors of the Medical and Physical Journal

**Published:** 1813-02

**Authors:** 


*To the Editors of the Medical and Physical Journal.*

GENTLEMEN,

THROUGH the medium of your useful Journal, I beg leave to call the attention of medical men to the treatment of concussion of the brain: the dissimilarity of treatment recommended by different authors, causes me to wish, that a certain rule could be given to the young practitioner, for his conduct, when accidents of this kind fall under his care.

If a person has received a blow upon his head, which has not fractured the skull, yet has deprived him of knowledge, caused restlessness, quick weak pulse, and a train of symptoms denoting convulsive action; the propriety of general bleeding is, I think, questionable;* I should therefore feel obliged if my wavering opinion could be rendered more firm, by the dispassionate reasoning of any of your correspondents.

I remain, Gentlemen,

Your most obedient Servant,

